# Histological Disorganization of Spleen Compartments and Severe Visceral Leishmaniasis

**DOI:** 10.3389/fcimb.2018.00394

**Published:** 2018-11-13

**Authors:** Micely d'El-Rei Hermida, Caroline Vilas Boas de Melo, Isadora dos Santos Lima, Geraldo Gileno de Sá Oliveira, Washington L. C. dos-Santos

**Affiliations:** Fundação Oswaldo Cruz, Instituto Gonçalo Moniz, Salvador, Brazil

**Keywords:** visceral leishmaniasis, white pulp disruption, spleen disorganization, *Leishmania infantum*, spleen pathology

## Abstract

The spleen is a secondary lymphoid organ responsible for immune surveillance against blood-circulating pathogens. Absence of the spleen is associated with increased susceptibility to systemic spread and fatal infection by different pathogens. Severe forms of visceral leishmaniasis are associated with disorganization of spleen compartments where cell interactions essential for splenic immunological function take place. White pulp atrophies, secondary lymphoid follicles and marginal zones vanish, and the boundaries separating white and red pulp blur. Leukocyte populations are reduced or disappear or are replaced by plasma cells. In this paper, we review the published data on spleen disorganization in severe forms of visceral leishmaniasis and propose a histological classification to help the exchange of information among research groups.

## Introduction

Visceral leishmaniasis (VL) is a severe parasitic disease caused by infection by *Leishmania infantum* (syn. *Leishmania chagasi*) or *Leishmania donovani* that affects both humans and dogs. Visceral leishmaniasis is distributed in Central and South America, Asia, parts of Africa and the Mediterranean basin, with an estimated burden of 2.1 million DALY (disability adjusted life years) (Townson et al., 2005). The infection may be silent or present with slight clinical manifestations (Badaró et al., 1986). However, some patients progress to a clear pattern of clinical disease with weight loss, hepatomegaly, splenomegaly, anemia, thrombocytopenia and leukopenia, including neutropenia, and increased susceptibility to bleeding and coinfections (Badaró et al., 1986; Costa et al., 2010). The current therapeutic regimes with antimonials or with amphotericin are effective in most of the cases of VL (Herwaldt, 1999). However, the disease maintains an approximately 7% lethality in Brazil, even among patients under treatment (Ministério da Saúde do Brasil, 2010).

The spleen, bone marrow, and liver are the main organs involved in VL. The spleen is affected in all cases of the disease. Furthermore, while some control of infection is observed in the liver, the infection acquires a progressive character in the spleen throughout the course of the disease (Wilson and Streit, 1996; Carrión et al., 2006), leading to disruption of white pulp (WP) structure and replacement of normal cellularity of the red pulp (RP) by plasma cells (Silva-O'Hare et al., 2016). Such structural disorganization of the spleen is associated with severe forms of VL (Veress et al., 1977; Lima et al., 2014). In this work, we review the published data on spleen disorganization in VL and the pathways involved in the process. Our aim is to draw attention to the spleen as a potential source of biological markers for identifying patients susceptible to progress to severe forms of VL. Early identification of these patients may contribute for designing more effective therapeutic strategies for progressive forms of the disease.

## The normal spleen

**Figure 1:**
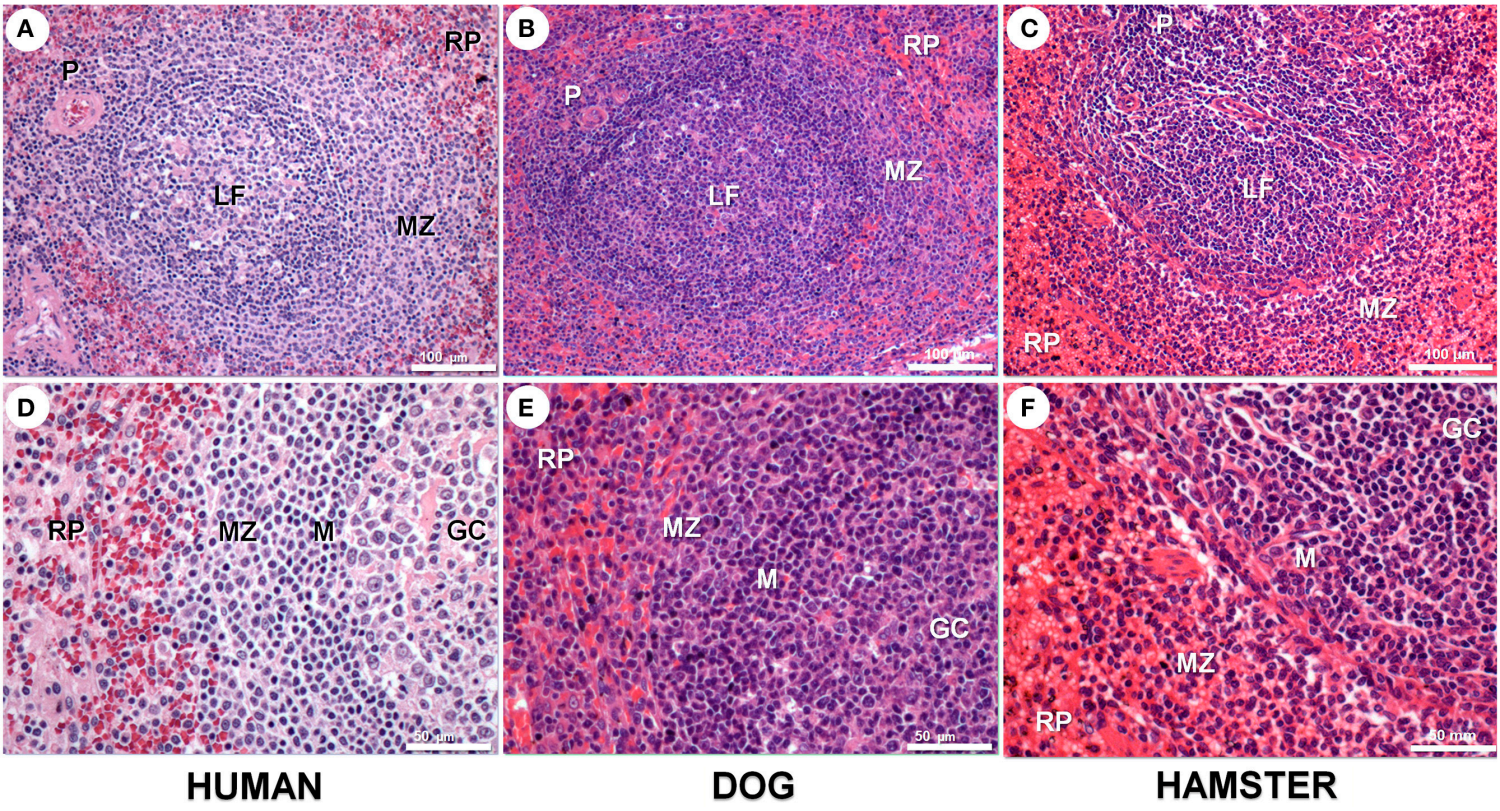
Spleen compartments in human **(A,D)**, dog **(B,E)**, and hamster **(C,F)** spleen. Spleen in all three species presents white and red pulp (RP). White pulp presents periarteriolar lymphocyte sheath (P) and lymphoid follicles with a germinal centers (GC) and mantle region (M) surrounded by a loosely distributed marginal zone (MZ). These spleen compartments are more clearly seen in human and dogs than in rodent spleens. Hematoxilyn-eosin staining, scale bar in **(A–C)** = 100 μm; in **(D–F)** = 50 μm.

The spleen is a large secondary lymphoid organ composed of two compartments: the RP and the WP, and present morphological variation among different species (Figure 1; Steiniger and Barth, 2000; Cesta, 2006). Cords, sinuses and blood vessels mainly comprise the RP, which contains lymphocytes macrophages, erythrocytes and a small number of plasma cells. The splenic RP perform hemocateresis and keeps strict control of iron stores, reducing their availability to circulating pathogens (Mebius and Kraal, 2005). The process involves the SLC11/Nramp (natural resistance-associated macrophage protein) family whose polymorphism is associated with susceptibility to a variety of pathogens (Wessling-Resnick, 2015). The spleen is the site of differentiation and homing of inflammatory macrophages, monocytes, granulocytes, dendritic cells, natural killer cells and short-lived plasma cells (Ellyard et al., 2005; Mebius and Kraal, 2005). In the WP, take place T- and B-cell differentiation and immune responses to blood-borne antigens (Mebius and Kraal, 2005). The WP is constituted by three regions: the periarteriolar lymphocyte sheath (PALS), lymphoid follicles and marginal zones (MZ). Layers of predominantly CD3^+^ T lymphocytes surrounding segments of small arteries forms the PALS. Contiguous with the PALS, the lymphoid follicles emerge as sparse round aggregates of predominantly B lymphocytes. There is always a variety in the primary and secondary lymphoid follicles. Primary lymphoid follicles are small nodular aggregates of small lymphocytes (Beyer and Meyer-Hermann, 2008). Secondary lymphoid follicles are large lymphoid aggregates that present germinal centers (GC). Germinal centers are composed of large proliferating lymphocytes, large macrophages sometimes containing apoptotic bodies, some follicular dendritic cells and some T lymphocytes (MacLennan, 1994). A ring of small lymphocytes (mantle zone) surrounds the follicle GC (Brozman, 1985). A diffuse layer containing predominantly B lymphocytes, some T lymphocytes and various macrophage populations form the MZ, which is more evident around the lymphoid follicles. The splenic MZ is the homing site for memory B cells responsive to T-lymphocyte dependent and T-lymphocyte independent response antigens (Kraal, 1992; Lopes-Carvalho et al., 2005).

The maintenance and organization of splenic compartments are controlled by a complex signaling network of chemokines (mostly of CC and CXC family), cytokines and adhesion molecules (den Haan et al., 2012). Lymphoid follicle architecture is dependent on CXCL13 secretion by stromal and follicular dendritic cells (Shi et al., 2001). CXCL13 interacts with the CXCR5 receptor on B cells recruiting these lymphocytes into the lymphoid follicle (Ansel et al., 2000). Lymphotoxin-α1β2 (LT-α1β2), produced by stromal cells, plays a crucial role in the early organization of the spleen (Fu and Chaplin, 1999; Mebius and Kraal, 2005). CCL21 is involved in the recruitment and retention of T lymphocytes in the PALS (Förster et al., 1999; Gunn et al., 1999). CXCL12 is responsible for plasma cell retention in the RP (Hargreaves et al., 2001). B-cell subtypes respond to antigens in a T-cell dependent (TD) manner. They enter the lymphoid follicle, interact with CD4^+^ T cells that express CD40 ligand in the germinal centers and differentiate into IgM-, IgG- or IgA-producing plasma cells with a high-affinity to antigens. Conversely, other splenic MZ B cells may be stimulated by B cell-activating factor (BAFF) and a proliferation-inducing ligand (APRIL) in a T-cell independent (TI) response, which plays an important role against microbial antigens (Bernasconi et al., 2002; Tsuji et al., 2008; Cerutti et al., 2011). The two pathways (TI and TD) are complementary to provide a more specific and faster diversified immune response (Cerutti et al., 2011; Grant et al., 2012).

## Spleen in non-infectious chronic diseases

Splenic structure and function are affected in the course of many chronic diseases. Long-lasting heart failure or impairment of liver circulation may lead to spleen congestion and stromal cell hyperplasia (Pereira et al., 2002). Hemoglobinopathies frequently course with splenic alterations (Tincani et al., 1997; O'Reilly, 1998). For example, in patients with sickle cell disease, the RP appears enlarged, with high numbers of lymphocytes and nucleated red blood cells (Szczepanek et al., 2012). Erythrocyte clumping, blood vessel obstruction and infarcts may lead to fibrosis and autosplenectomy (Al-Salem, 2011). Splenomegaly has been reported in approximately 9–41% of hepatic diseases, 4–10% of congestive or inflammatory diseases, 16–44% of lymphomas (Pozo et al., 2009) and 3% of the cases of sarcoidosis (Judson, 2007). Lymphoid follicle hyperplasia is found in systemic lupus erythematous (SLE) and other autoimmune diseases (Auerbach et al., 2013). In SLE, polyclonal B-cell activation results in an increased number of immunoblasts, plasmacytoid lymphocytes and plasma cells in the RP (Mok and Lau, 2003). Spleen arterioles develop a hyperplasic onion-skin aspect, composed of multiple layers of fibrosis and smooth muscle cell proliferation (Kitamura et al., 1985). In a series of SLE cases, extramedullary hematopoiesis was observed (Auerbach et al., 2013). In late stage diseases, lymphoid atrophy may follow (Li et al., 2013).

## The spleen and infection

The spleen plays a central role in defense against circulating pathogens. Absence of the spleen predisposes to devastating consequences with the dissemination of infections by viruses, bacteria and fungi (Hansen and Singer, 2001). Changes in spleen structure are common in many systemic infections caused by viruses, bacteria and parasites (Andrade et al., 1990; Kyaw et al., 2006). Some of these infections progress with lymphoid or stromal splenic cell hyperplasia, sometimes followed by lymphoid atrophy and disorganization of spleen compartments (Abreu et al., 2001; Sonne et al., 2009; Schneider et al., 2010; Glatman Zaretsky et al., 2012; Dkhil et al., 2014; Djokic et al., 2018).

Viral infections such as Mononucleosis cause splenomegaly with mononuclear cell proliferation and atypical lymphocytes (Daneshbod and Liao, 1973; Thomas et al., 1990; Won and Ethell, 2012). In patients with AIDS, there is a progressive destruction of FDCs and concomitant germinal center loss (Fox and Cottler-Fox, 1992). Parvovirus infection in dogs causes splenomegaly, with lymphoid follicle hyperplasia, bleeding foci and congestion in the splenic parenchyma (Oliveira et al., 2009). In the course of bacterial infection acute splenitis, septic emboli originating, leading to infarcts and abscesses may occur (Mocchegiani and Nataloni, 2009; Wang et al., 2009). Infection with hemoparasites such as *Plasmodium, Ehrlichia, Babesia, Toxoplasma gondii* are all associated with splenomegaly, lymphoid tissue hyperplasia and eventually to disruption of WP structure. *Schistosoma mansoni* infection causes congestive splenomegaly and decreased number of lymphoid follicles and blurred marginal zones (Andrade and Bina, 1983; Brandt et al., 2005; Silva et al., 2012; Wang et al., 2015; Yan-Juan et al., 2017).

## Structural disorganization of the spleen compartments

Most of the studies concerning the morphological changes of the spleen occurring in the course of infectious and non-infectious diseases have emphasized the quantitative aspect of lymphoid or stromal hyperplasia or atrophy. Only a few authors have drawn attention to the association of the morphological changes with the redistribution of leukocyte populations resulting in the remodeling of splenic microenvironments in the course of infection and inflammation. Veress et al. (1977) first used the term disorganization to describe the changes affecting the spleen compartments in patients who died of VL. The authors considered that the change in leukocyte populations with depletion of T lymphocytes would lead to impairment of the cell interactions necessary to kill the parasite (Veress et al., 1977). Morrison et al. (1981b), studying *Trypanosoma brucei* infection, also reported a lack of reactive germinal centers and a disarrangement of lymphoid follicles in late stages of the disease. The authors considered these alterations an indication of poor function of the lymphoid system (Morrison et al., 1981b). In fact, a general description of loss of the normal architecture of the spleen is found in many studies. However, a precise definition of the histological parameters changed by infectious diseases is frequently lacking. In the studies where a more detailed description of spleen changes is presented and hyper- or hypo-plastic alterations of the white and RP of the spleen is described, an MZ effacement and macrophage emigration from the MZ into the white and RP are reported (Table 1). Therefore, the term disorganization is applied to the spleen for a number of distinct changes of spleen compartments.

**Table 1:** Published papers mentioning spleen histological disorganization associated with parasitic infections.

**References**	**Parasite**	**Host**	**Histological observations**
Dash et al., 2005	*Dermacentor andersoni*	Mouse	“[…] disruption of the white-pulp/red-pulp demarcations and the presence of a large number of basophilic normoblasts.”
Veress et al., 1977	*Leishmania donovani*	Human	“[…]in none of the twenty 20 cases of kala-azar studied were any germinal centers seen and the white pulp itself was loosened and disorganized. In six out of the twenty 20 cases center necrosis was found destroying the normal architecture of the white pulp. The number of lymphocytes was invariably very low in all white pulps examined; and occasionally, the lymphocytes were virtually missing, being replaced by plasma cell and parasite-containing histiocytes.”
Santana et al., 2008	*Leishmania infantum*	Dog	“Degree of structural organization of the white pulp, in which the white pulp was classified as: well organized, with distinct peri-arteriolar lymphocyte sheath, germinal center, mantle zone and marginal zone; slightly disorganized, with either hyperplastic or hypoplastic changes leading to a loss in definition of any of the regions of the white pulp; moderately disorganized, when the white pulp was evident, but its regions were poorly individualized or indistinct; extensively disorganized, when the follicular structure was barely distinct from the red pulp and T-cell areas. The last two categories were frequently associated with lymphoid atrophy.”
Tasca et al., 2009	*Leishmania infantum*	Dogs	“[…] diffuse chronic inflammation with thickness of capsular and trabecular regions and […] morphological alteration of the red and white pulp by the presence of abundant macrophages filled with amastigotes, the granulomatous inflammatory reaction and hemorrhagic areas.”
de Lima et al., 2012	*Leishmania infantum*	Dog	“(1) slightly disorganized, with either hyperplastic or hypoplastic changes leading to a loss of definition of any of the regions of the white pulp and (2) for moderately or extensively disorganized, when the white pulp regions were poorly individualized or indistinct.”
Silva et al., 2012	*Leishmania infantum*	Dog	The authors use a similar description as Santana et al. (2008) adding: “[…] These changes in the white pulp were associated with decreased numbers of T and dendritic cells in the follicles and B cells in the follicles and marginal zones.”
Lima et al., 2014	*Leishmania infantum*	Dog	The authors use a similar description as Santana et al. (2008) and group moderately and severely disorganized spleen into a single class type 3 spleen “[…] spleen type 3 (moderately to extensively disorganized) has white pulp evident but with poorly individualized or indistinct regions or in which the follicular structure was barely distinct from the red pulp and T-cell areas. The last category is frequently associated with lymphoid atrophy.”
Cavalcanti et al., 2015	*Leishmania infantum*	Dog	“[…] as described by Santana et al. (2008).”
Silva-O'Hare et al., 2016	*Leishmania infantum*	Dog	“[…] as described by Lima et al. (2014).”
da Silva et al., 2018	*Leishmania infantum*	Dog	The authors use a similar description as Santana et al. (2008) and add: “The deposition of the extracellular matrix components is associated with the disorganization of the splenic white pulp; Metallopeptidase-9 expression is higher in dogs with disorganized splenic white pulp than in dogs with organized spleen; The CD4^+^ cell number is lower in the disorganized spleen than in the organized spleen.
Morrison et al., 1981a	*Trypanosoma congolense*	Mouse	“Following the initial proliferative changes, a more protracted phase ensued during which, although the proliferative activity continued, there was a gradual disorganization of the white pulp with eventual lymphoid depletion. This was accompanied by a progressive expansion of the red pulp due to increased numbers of erythropoietic cells and to a lesser extent granulopoietic cells and macrophages. At the same time, there was a gradual decrease in the number of plasma cells found in the red pulp, although many were still present in the periarteriolar regions.”
Morrison et al., 1981b	*Trypanosoma brucei*	Dog	“Following the initial proliferative phase and prior to the death of the host during the fourth week of the infection, the spleen […] became less reactive, and there was marked disorganization and disruption of their architecture. […] germinal centers were reduced in number, size, and activity, had a disorganized appearance […].”
Krucken et al., 2005	*Plasmodium chabaudi*	Mouse	“[…] marginal zone macrophages (MZM) were lost and red pulp macrophages entered the white pulp.”
Al-Shaebi et al., 2017	*Plasmodium chabaudi*	Mouse	“The infection causes disorganization of macrophage distribution in the spleen.”
Keswani and Bhattacharyya, 2013	*Plasmodium berghei*	Mouse	“[…] cells in the white pulp had proliferated considerably and enlarged to the limits wherein the margin between white and red pulp began to disappear and hollow spaces without cells appeared. Follicle germinal centers (GC) lost the typical architecture acquiring a disorganized aspect […].”
Urban et al., 2005	*Plasmodium falciparum*	Human	“ […]The red pulp was frequently congested […]; […] profound depletion of B cells from the marginal zone […]; […] frequency of germinal centers within lymphoid follicles was significantly reduced […].”
Alves, 2015	*Plasmodium falciparum*	Monkey	“[…] spleens showed disruption of germinal center architecture with heavy B-cell activation (centroblasts). During the acute phase of infection, splenic disarray with disorganized germinal centers was observed.”
da Silva et al., 2012	*Schistosoma mansoni*	Mouse	“[…] splenic disorganization such as a decrease in the numerical density of white pulp and, red pulp and germinal center hyperplasia.”
Bauomy et al., 2014	*Schistosoma mansoni*	Mouse	“[…] infection induced splenomegaly and the spleen appeared with disorganized red and white pulps […].”

## Spleen disorganization in VL

Although spleen changes are reported in many diseases, there are few systematic studies about the disruption of spleen compartments. We performed a systematic search in PubMed, Web of Science and SCOPUS databases using the following keywords: spleen, disruption, disorganized, disorganization and white pulp. The search resulted in a total of 17 articles. Nine of these articles mentioned *Leishmania* infection (Table 1).

The spleen presents sequential changes during the course of VL in all susceptible hosts. The most evident change is spleen enlargement. In humans, the spleen may reach the right lower quadrant of the abdomen and gives rise to hypersplenism, a syndrome characterized by anemia, thrombocytopenia and low white blood cell counts (Dos-Santos et al., 2014). In fact, a decrease in spleen size is used as a parameter of therapeutic response in human VL (Kip et al., 2015). *Leishmania*-infected macrophages are observed in all spleen compartments (Andrade and Andrade, 1966). However, parasite distribution and parasite load are heterogeneous in the spleen. Culture of fine needle aspirate collected from different areas of the spleen of dogs with VL showed a variation of 3.2 and 34.7 folds in parasite load present in different regions of the upper, middle and lower third of the organ (Bagues et al., 2018). Associated granulomas, perisplenitis and progressive changes of leukocyte populations are frequently observed (Santana et al., 2008). Initially, the WP shows hyperplastic reactive lymphoid follicles and an increased number of macrophages (Veress et al., 1983; Keenan et al., 1984; Bamorovat et al., 2014). As the disease progresses, reactive lymphoid follicles are disrupted, sometimes replaced by hyaline deposits (Veress et al., 1983). The usual boundaries between the WP compartments are progressively effaced, the mantle zone progressively vanishes, and the MZ becomes less evident (Veress et al., 1977). In chronic severe forms of the disease, the spleen presents WP atrophy, to the degree that secondary lymphoid follicles are no longer found (Santana et al., 2008; Santos et al., 2016). A clear distinction between white and RP is not always feasible and numerous plasma cell aggregates replace the normal resident cell populations of the RP (Santana et al., 2008; Silva-O'Hare et al., 2016). Therefore, the increase in the size and redistribution of cell populations corresponds to an advanced state of functional disorganization of the spleen both hematologically and of defense against infections. These changes may impair the host ability to respond to infection by *Leishmania* and other pathogens.

**Figure 2:**
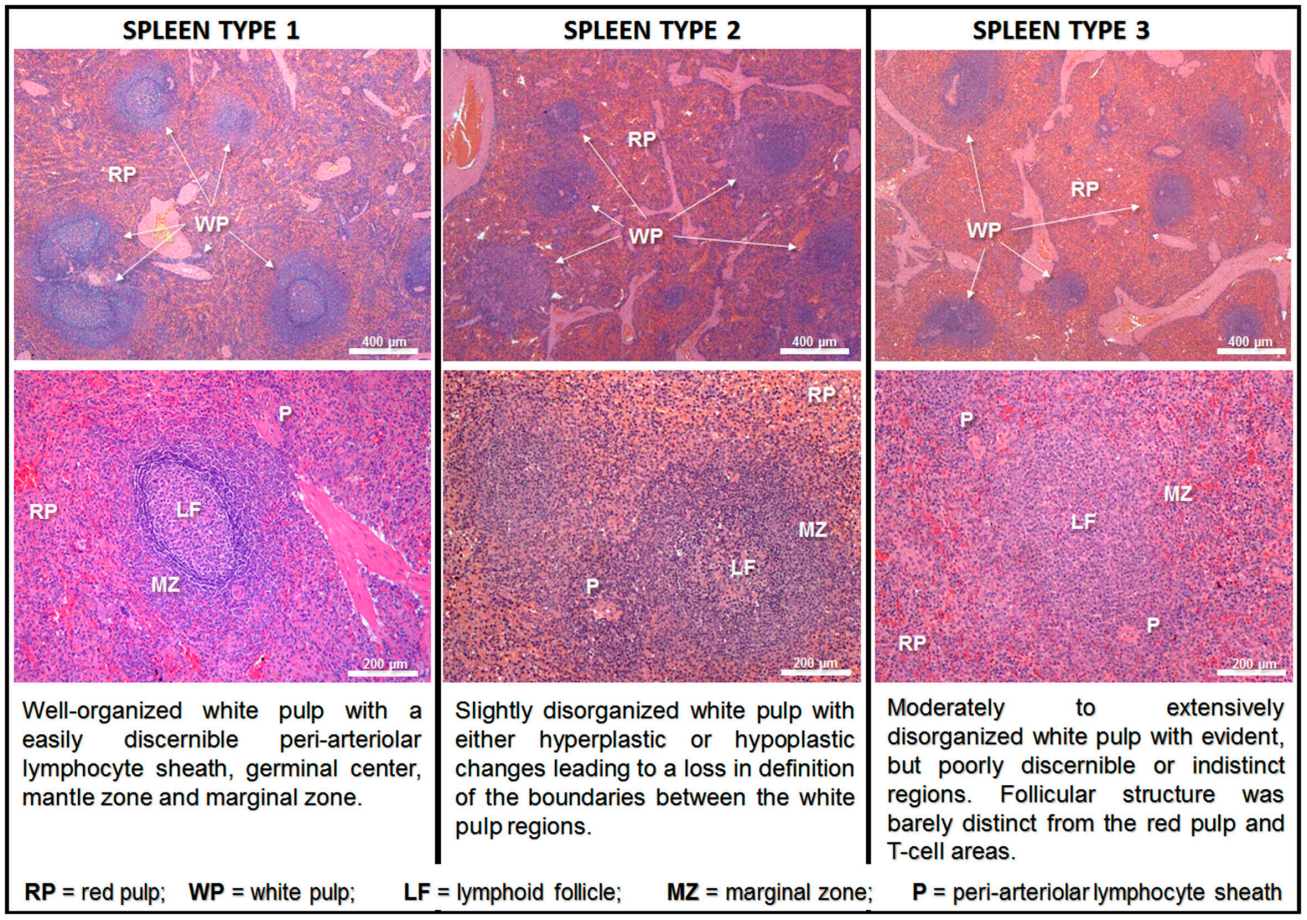
Class of spleen disorganization according to disruption of white pulp structures. Hematoxilyn-eosin staining, scale bar in the top row = 400 μm; in the bottom row = 200 μm.

To allow a better exchange of information between groups working with the structural changes of the spleen, we proposed a morphological classification of splenic WP organization based on the level of disruption of the different compartments. This classification was based on consensus analysis of the spleens of 72 dogs from an endemic area of VL performed by three pathologists. Initially, four categories were proposed: normal, slightly disorganized, moderately disorganized and intensely disorganized (Santana et al., 2008). In subsequent studies, to improve the agreement between observers, we maintained three categories, collapsing the moderately and intensely disorganized categories into a single category (Figure 2). Well organized spleen (spleen type 1) has a distinct periarteriolar lymphocyte sheath, lymphoid follicles and a marginal zone. Present a varied number of secondary lymphoid follicles, containing a germinal center clearly delimited by a rim of small lymphocytes (the mantle zone); slightly disorganized (spleen type 2) has either hyperplastic or hypoplastic changes blurring the boundaries between regions of the white pulp; and moderately to extensively disorganized (spleen type 3) the white pulp regions are poorly individualized or indistinct lymphoid follicles are barely distinct from the red pulp and T-cell areas and secondary lymphoid follicles are absent. As shown in Figure 2, the type 1 and type 3 spleens are polar categories of easily distinguished organization and disorganization of the WP. All the cases that do not fit into these two categories are classified as type 2 spleen (Silva-O'Hare et al., 2016). In a survey of stray dogs collected from an endemic area of VL, 23% (48/206) of the semi-domiciled animals, most of them with *Leishmania* infection, presented type 3 spleens (Lima et al., 2014). Morphometric studies showed that animals with active leishmanial infection confirmed by serology or spleen culture and type 3 spleen had a smaller WP/RP proportion (6%) than animals with type 1 spleen (13%) (Silva et al., 2012). The decrease in WP size affects predominantly the lymphoid follicles and the MZs, which are 3.5 and 1.9 times smaller, respectively, in animals with type 3 than those of animals with type 1 spleen (Silva et al., 2012). CD79α+ B lymphocytes were decreased in the lymphoid follicles in the MZ and CD3+ T lymphocytes were decreased in lymphoid follicles (Silva et al., 2012). Additionally, da Silva et al. (2018) showed a decrease in number of CD4^+^ T lymphocytes and de Lima et al. (2012) showed increased T-cell apoptosis in the disrupted white pulp of naturally infected dogs with VL (de Lima et al., 2012; da Silva et al., 2018). These observations suggest that T-cell exhaustion may play a role in the progression of splenic alterations.

These changes in the spleen are associated with the parasite burden and with a decrease in mRNA expression of pro-inflammatory and anti-inflammatory cytokines related to the immune response to *Leishmania* such as IFNγ, IL-12, IL-6, TNF, IL-10, and TGFβ (Cavalcanti et al., 2015). Follicular dendritic cells, an important source of CXCL13, are also decreased in lymphoid follicles (Silva et al., 2012). CXCL13 is a chemokine responsible for B-cell migration into the lymphoid follicle and in lymphoid follicle maintenance (Neely and Flajnik, 2015). Smelt et al. (1997) suggested that *Leishmania*-infected cells present in the lymphoid follicles may correlate with follicular dendritic cell death, which may explain their reduction in late stages of the disease (Smelt et al., 1997). In fact, the gene expression for CXCL13 is significantly decreased in the spleens of dogs with active *L. infantum* infection and type 3 spleen (Silva et al., 2012). Furthermore, changes in the extracellular matrix components such as laminin and collagen, as well as an increased expression of Metallopeptidase-9 may also participate in the process (da Silva et al., 2018).

**Figure 3:**
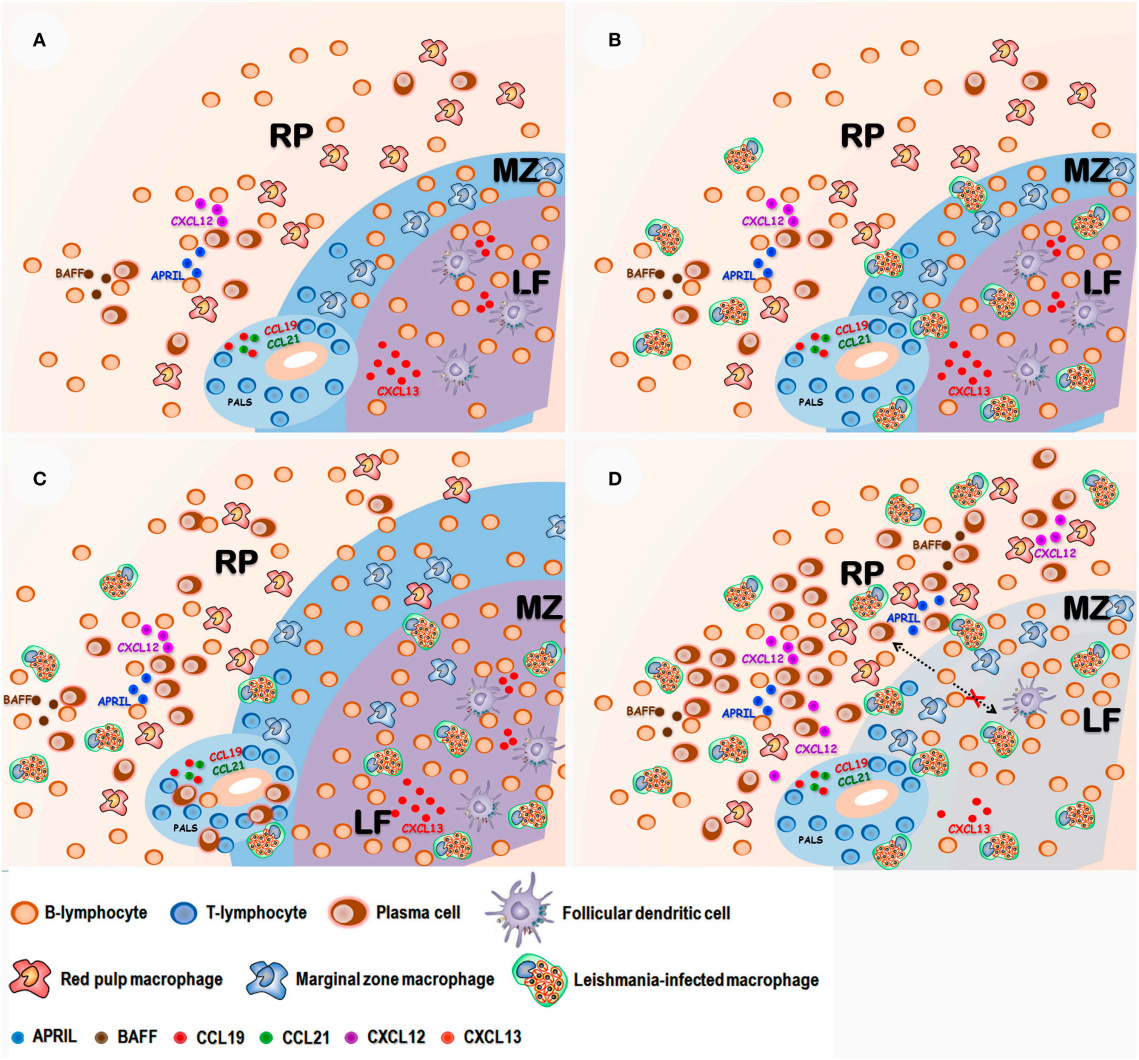
Proposed sequence of events in spleen disorganization: **(A)** Normal spleen with well-defined white and red pulp compartments: Red pulp (RP) with a mixed leukocyte population with lymphocytes, macrophages and some plasma cells. Marginal zone (MZ) containing lymphocyte and macrophages. Lymphoid follicle (LF) containing lymphocytes and follicular dendritic cells. Periarteriolar lymphocyte sheath (PALS) containing mostly predominantly lymphocytes. The integrity of spleen compartments is dependent on chemokines such as CCL19 and CCL21 (PALS), CXCL13 (LF). **(B)** After *Leishmania* infection amastigote-containing macrophages are observed in the different spleen compartments. **(C)** Antigen stimulation and polyclonal B cell activation leads to white pulp hyperplasia and plasma cell accumulation in the RP. **(D)** T lymphocyte and follicular dendritic cell apoptosis leads to a reduction of CXCL13 chemokine expression and white pulp disruption. Inflammatory changes of red pulp enhance B-cell activating factor (BAFF), A proliferation-inducing ligand (APRIL) and CXCL-12 chemokine expression, favoring plasma cells homing and survival.

Important changes in cell populations are also observed in splenic RP. Plasma cells progressively become the more frequent leukocyte present in this compartment (Santana et al., 2008; Silva-O'Hare et al., 2016). Plasma cells are highly specialized antibody-secreting cells derived from B lymphocytes. These cells have a complex biology arising from follicular or extrafollicular B-lymphocyte differentiation. Most plasma cells are short lived, remaining only for a few days in the WP in the spleen. Some plasmablasts migrate to the bone marrow, where a few differentiate to long-lived plasma cells (Tangye, 2011). Unfortunately, little is known about the role played by these cells in chronic inflammatory infiltrates. They are present in the spleen and are an important cell component of inflammatory infiltrates found in different organs of patients with VL (Andrade and Andrade, 1966). Spleen plasmacytosis correlates with the dysproteinemia presented by dogs with VL (Silva-O'Hare et al., 2016). Most of the plasma cells that accumulate in the spleen in VL are IgG-producing cells. The RP plasmacytosis persists after WP disorganization and complete disruption of lymphoid follicles (Silva-O'Hare et al., 2016). This finding suggests that most of these cells continue to accumulate in the spleen either by extra follicular B-cell differentiation or by an increased life span in the RP. In fact, BAFF, APRIL and CXCL12 cell factors involved in plasma cell homing and survival are increased in the spleen of dogs with active VL and type 3 spleen (Silva-O'Hare et al., 2016). This observation suggests that inflammatory changes in the RP microenvironment may favor an increase of life span of plasma cells resulting in a shift in the composition of the leukocyte population in this spleen compartment (Figure 3).

Spleen disorganization is associated with more severe VL presentations. Dogs with active infection by *L. infantum* and type 3 spleen had more frequent clinical signs of disease (alopecia, anemia, conjunctivitis, dehydration, emaciation, onychogryphosis, skin erosion and ulceration), more frequently altered laboratory biochemistry and hematological tests (lower serum albumin, higher serum AST, decreased red blood cell counts and increased number of neutrophils) and more frequently negative leishmanin skin test than animals with active *Leishmania* infection and type 1 spleen (Lima et al., 2014; da Silva et al., 2018).

In humans who died with severe VL, Veress et al. (1977) found WP atrophy and disorganization, absence of GC-containing lymphoid follicles, decrease in lymphocyte number and lymphocyte replacement by plasma cells (Veress et al., 1977). Recently, we comparatively studied the spleens of patients who died with VL with the spleens of patients who died of other chronic diseases and observed that patients who died of VL had more frequent WP disruption than patients without VL (unpublished data). Furthermore, patients with VL present increased levels of BAFF a cytokine involved in plasma cell differentiation and survival in the serum (Goto et al., 2014). These data suggest that the disorganization of splenic microenvironments is also relevant to human disease. In fact, sporadic reports suggest that splenectomy may contribute to the cure of patients with chronic relapsing human VL resistant to conventional drug treatment (Rees et al., 1984; Alon and Chowers, 2012).

## The relevance of spleen changes in VL

In this review, we draw attention to the marked changes in the organization of spleen compartments in severe forms of VL. These changes combine an increase in the size and altered distribution of cell components. The cell populations undergo change, and the appropriated sites of cell interactions and differentiation disappear or are substantially disrupted. Secondary lymphoid follicles, a site of development and refinement of antibody immune response, are no longer observed and MZs, homing sites for a variety of memory B cells are almost nonexistent. Since the spleen changes are usual in the whole course of VL, we may consider the role played by the disease in the genesis of these alterations. Furthermore, these changes in the spleen are associated with severe/terminal disease in dogs and in humans. Given the role of spleen in the protection against bloodborne pathogens the change in cell postulations and cytokine expression pattern in disorganized spleen may contribute to the progression of the disease by increasing host susceptibility to *Leishmania* and other pathogens. In fact, bacterial infection is among the main causes of death in patients with VL (Andrade et al., 1990; Costa et al., 2010). Therefore, the changes observed in the white and RP may impair or subvert the normal immunological functions of the spleen. A better understanding of the sequence of events and pathways involved in spleen disorganization may help to develop more sensitive tests for detecting progressive forms of VL and to design new approaches to the treatment of the disease.

## Author contributions

WD-S, GO, MH conceived the manuscript. MH, CM, IL wrote the text. WD-S, GO revised the text. WD-S, MH, produced the figures.

### Conflict of interest statement

The authors declare that the research was conducted in the absence of any commercial or financial relationships that could be construed as a potential conflict of interest.
